# Metabolites and Biomarker Compounds of Neurodegenerative Diseases in Cerebrospinal Fluid

**DOI:** 10.3390/metabo12040343

**Published:** 2022-04-12

**Authors:** Keiji Wakamatsu, Yoichi Chiba, Ryuta Murakami, Yumi Miyai, Koichi Matsumoto, Masaki Kamada, Wakako Nonaka, Naoya Uemura, Ken Yanase, Masaki Ueno

**Affiliations:** 1 Department of Pathology and Host Defense, Faculty of Medicine, Kagawa University, Miki-cho 761-0793, Japan; s20d727@kagawa-u.ac.jp (K.W.); chiba.yoichi@kagawa-u.ac.jp (Y.C.); murakami.ryuta@kagawa-u.ac.jp (R.M.); miyai.yumi@kagawa-u.ac.jp (Y.M.); matsumoto.koichi@kagawa-u.ac.jp (K.M.); 2 Department of Neurological Intractable Disease Research, Faculty of Medicine, Kagawa University, Miki-cho 761-0793, Japan; kamada.masaki@kagawa-u.ac.jp; 3 Department of Neurology, Faculty of Medicine, Kagawa University, Miki-cho 761-0793, Japan; nonaka.wakako@kagawa-u.ac.jp; 4 Department of Anesthesiology, Faculty of Medicine, Kagawa University, Miki-cho 761-0793, Japan; uemura.naoya@kagawa-u.ac.jp (N.U.); yanase.ken@kagawa-u.ac.jp (K.Y.)

**Keywords:** biomarker, cerebrospinal fluid, choroid plexus, epithelial cell, tight junction, microRNA

## Abstract

Despite recent advances in diagnostic procedures for neurological disorders, it is still difficult to definitively diagnose some neurodegenerative diseases without neuropathological examination of autopsied brain tissue. As pathological processes in the brain are frequently reflected in the components of cerebrospinal fluid (CSF), CSF samples are sometimes useful for diagnosis. After CSF is secreted from the choroid plexus epithelial cells in the ventricles, some flows in the brain, some is mixed with intracerebral interstitial fluid, and some is excreted through two major drainage pathways, i.e., the intravascular periarterial drainage pathway and the glymphatic system. Accordingly, substances produced by metabolic and pathological processes in the brain may be detectable in CSF. Many papers have reported changes in the concentration of substances in the CSF of patients with metabolic and neurological disorders, some of which can be useful biomarkers of the disorders. In this paper, we show the significance of glucose- and neurotransmitter-related CSF metabolites, considering their transporters in the choroid plexus; summarize the reported candidates of CSF biomarkers for neurodegenerative diseases, including amyloid-β, tau, α-synuclein, microRNAs, and mitochondrial DNA; and evaluate their potential as efficient diagnostic tools.

## 1. Introduction

**Figure 1:**
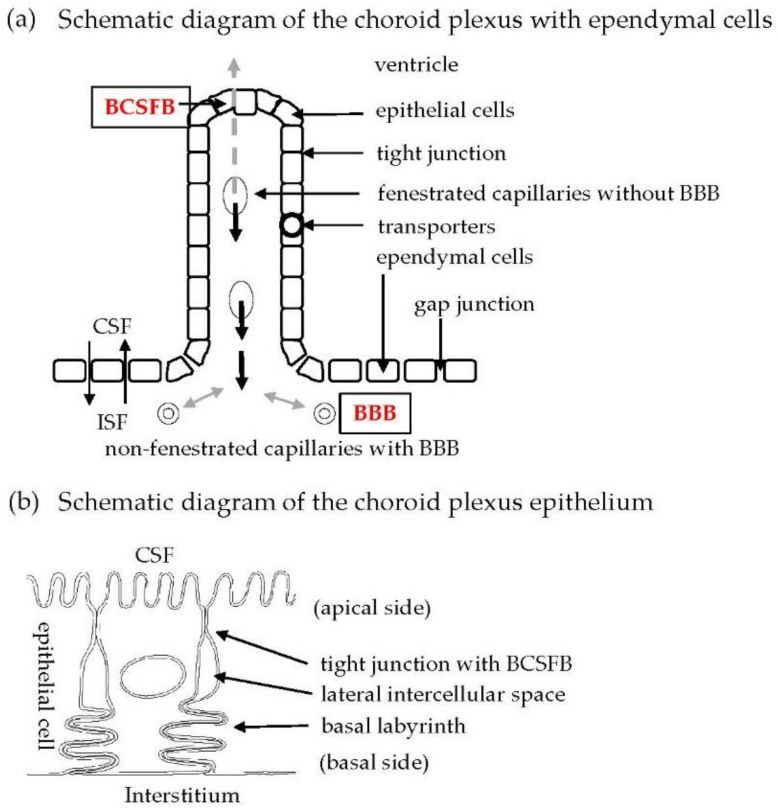
(**a**) Schematic representation of BCSFB in CPE cells. Fenestrated capillaries are located in the stroma of CP. CPE cells facing the ventricle are bound by tight junctions. Ependymal cells mainly bound by gap junctions are located between the ventricle and brain parenchyma. Transporters are localized in the cytoplasmic membrane of CPE cells. Non-fenestrated capillaries with tight junctions between endothelial cells are situated in the brain parenchyma and have a tight barrier function, referred to as the blood–brain barrier (BBB). (**b**) Schematic diagram showing localization of the tight junction, lateral intercellular space, and basal labyrinth on the lateral side of CPE cells.

Cerebrospinal fluid (CSF) is produced by the choroid plexus (CP), which is situated in the lateral, third, and fourth ventricles. CP with a vascularized stroma is covered by a monolayer of epithelial cells interconnected by tight junctions (Figure 1a) [1,2]. Although the endothelial cells of capillaries in the CP parenchyma are fenestrated, allowing the passage of intravascular macromolecules to the stroma, entry of the macromolecules into CSF is restricted by the neighboring epithelial cell layer (Figure 1a,b) [3,4]. Figure 1b shows the localization of the tight junction, lateral intercellular space, and basal labyrinth on the lateral side of choroid plexus epithelial (CPE) cells. These structures serve as a barrier between the blood and CSF, referred to as the blood–CSF barrier (BCSFB).

However, multiple ion transporters are localized on the apical and basal sides of the cytoplasmic membrane of CP epithelial (CPE) cells [4,5], as shown in Table 1. Accordingly, CP produces and secretes a major fraction of CSF [6]. Standard concentrations of major ionic compounds in the CSF of rabbits have been reported to be 149 mM Na^+^, 2.9 mM K^+^, and 130 mM Cl^−^, showing 305 mOsm/L and pH 7.27 [4]. There are some differences in the concentrations of several kinds of ions among species and in the literature, as shown in Table 2 [4,7,8]. Through multiple ion transporters, about 500 mL of CSF a day is secreted into ventricles in human adults [6]. Some CSF flows in the brain, is mixed with interstitial fluid (ISF), and is excreted to the venous system. Pathways for the return of CSF to the venous system are still being debated. CSF was considered to be reabsorbed directly through arachnoid granulations into the venous sinuses in the dura of the brain [9]. Recently, however, alternative pathways for CSF to return to the systemic circulation are considered to be via meningeal lymph vessels [10]. Intracerebral fluids have also been suggested to flow along peri- and para-vascular spaces surrounding cerebral arterial vessels and to drain into the venous system. The perivascular drainage route for ISF is known as the intravascular periarterial drainage (IPAD) pathway [11,12,13]. Another paravascular route for CSF is known as the glymphatic system [13,14,15]. The two major routes for the flow of CSF and ISF are believed to play a significant role in the discharge of wastes produced in the brain.

**Figure 2:**
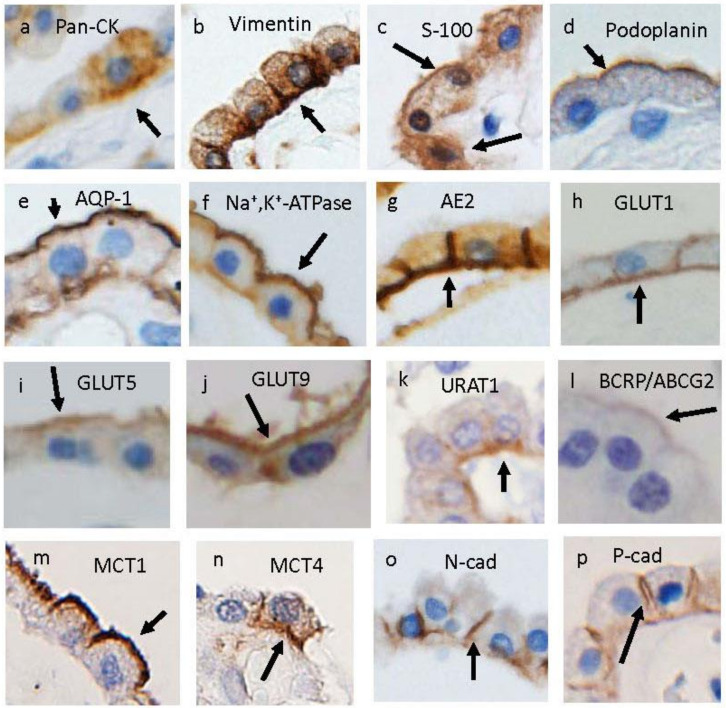
Localization of representative cytoplasmic membrane (**a**–**d**); anion/cation transporters (**e**–**g**); transporters for glucose (**h**), fructose (**i**), urate (**j**–**l**), and lactate (**m**,**n**); and junctional components (**o**,**p**) in autopsied human brains. Immunoreactivities for pan-cytokeratins (pan-CK) (**a**: DAKO, M3515); vimentin (**b**: DAKO, M0725); S-100 (**c**: Nichirei, 422091); podoplanin (**d**: DAKO, M3619); AQP-1 (**e**: ProteinTech, 20333-1-A); Na^+^,K^+^-ATPase (**f**: Santa Cruz, sc-48345); anion exchanger 2 (AE2) (**g**: SantaCruz, sc-376632); transporters for glucose (GLUT1) (**h**: Abcam, ab150299), fructose (GLUT5) (**i**: IBL, 18905), urate (GLUT9) (**j**: Abcam, ab104623), urate (URAT1) (**k**: MBL, BMP064), urate (BCRP/ABCG2) (**l**: Abcam, ab3380), lactate (MCT1) (**m**: Merck, AB3538P), and lactate (MCT4) (**n**: Abcam, ab244385); N-cadherin (**o**: ProteinTech, 22018-1-AP); and P-cadherin (**p**: Santa Cruz, sc-74545) are observed in the cytoplasm of luminal or basolateral cytoplasmic membrane of CPE cells.

CPE cells are characterized by positive immunoreactivities for cytokeratin, vimentin, S-100 protein, and podoplanin [16,17,18] (Figure 2a–d). CPE cells are equipped with several transporters for glucose-related substances as well as for CSF secretion in the apical and/or basolateral cytoplasmic membrane (Figure 2e–n). The well-known transporters in apical and basolateral cytoplasmic membranes, such as aquaporin 1 (AQP1), Na^+^, K^+^, ATPase, and anion exchange protein 2 (AE2), are shown in Figure 2e-g. In addition, transporters for glucose (Figure 2h), fructose (Figure 2i), urate (Figure 2j–l), and lactate (Figure 2m,n) are shown in Figure 2. It is also known that tight junctions between epithelial cells are mainly composed of occludins and claudins. N-cadherin (Figure 2o) and P-cadherin (Figure 2p) have been reported to be expressed in the lateral membrane and basal labyrinth of CPE cells [4,19], whereas it remains controversial whether the typical epithelial cadherin, E-cadherin, is expressed in the CPE cells of mammalian brains [4,20]. Claudin-1, -2, -3, and -11 are expressed in the tight junctions of CPE cells [21,22]. Interestingly, claudin-2 allows the unique transport permeation of monovalent cations, as well as H_2_O [23], and may contribute to the transepithelial resistance of CP.

The brain parenchyma and ventricles are separated by ependymal cells with gap junctions, whereas the brain and blood are separated by endothelial cells with tight junctions. Accordingly, it is likely that excess intracerebral waste products and metabolites without transporters for them can easily move into the ventricle rather than into the blood. Accordingly, we focused on metabolites and substances caused by neurodegeneration in CSF.

In this paper, we first review glucose-related CSF metabolites routinely analyzed within laboratory practice, such as glucose, fructose, urate, and lactate, and neurotransmitter (dopamine and serotonin)-related CSF metabolites. Second, we review amyloid-β-, phosphorylated tau protein-, and α-synuclein protein-related CSF compounds to identify the CSF biomarker candidates of neurodegenerative diseases. It is well known that amyloid-β and phosphorylated tau proteins deposit in the parenchyma of AD brains, whereas phosphorylated α-synuclein deposits in the brain parenchyma of patients with Parkinson’s disease. In addition, phosphorylated tau and/or α-synuclein proteins deposit in the brains of patients with several kinds of Parkinsonism, including tauopathy and synucleinopathy. Accordingly, these excessive proteins in the brain parenchyma were expected to migrate into the ventricle, possibly via the ependymal cell layer with a gap junction. In addition, post-synaptic proteins, such as neurogranin and neuroligin-1, and cytoskeletal proteins, such as neurofilament light chain, were also investigated in CSF as markers of synaptic or cytoskeletal dysfunction, occurring in the brains of patients with neurodegenerative diseases. Lastly, as it is gaining attention that intracellular nucleic acids, such as microRNA and mitochondrial DNA, migrate extracellularly and can be found in CSF, we review these substances in CSF as candidate biomarkers for neurodegenerative diseases.

## 2. Glucose- and Neurotransmitter-Related CSF Metabolites

### 2.1. Glucose-Related CSF Metabolites

#### 2.1.1. Glucose

The glucose level in the CSF of individuals with normoglycemia is 50–80 mg/dL (2.78–4.44 mM), which corresponds to approximately 50–60% of the plasma glucose concentration. The net glucose flux across CPE cells is considered to be 2–3% of those across cerebral vessels [24]. Basolateral-predominant expressions of glucose transporter 1 (GLUT1) (Figure 2h) and possibly sodium/glucose cotransporter 2 (SGLT2) [25] in CPE cells suggest their contribution to supplying CSF with glucose via CPE cells [1,24,26].

Several studies have demonstrated that CP shows age-associated morphological and functional alterations. Aged CPE cells exhibit reductions in the height, total volume, and length of apical villi, resulting in a more flattened appearance [3,27]. In addition, reduced CSF production in aged subjects has also been observed in humans, rats, and sheep [3,27,28]. Judging from the analysis results of CSF glucose concentrations in 8871 individuals, CSF glucose concentrations increase with aging [29]. In addition, marked increases in glucose concentrations in the CSF, as well as in the blood, of 25 patients with diabetes mellitus have been noted [8]. It is well known that, in CSF leukocytes, glucose, lactate, and proteins are commonly used as CSF markers of bacterial meningitis in routine care worldwide [30].

Concerning glucose metabolism, dynamic ^18^F-fluorodeoxyglucose (FDG) PET scans revealed that subjects (*n* = 17) with Alzheimer’s disease (AD) show reduced 18F-FDG metabolism in CP compared with subjects with amnestic mild cognitive impairment (*n* = 10) or healthy subjects (*n* = 20) [31]. This glucose metabolism imbalance in patients with AD may be attributed to reduced glucose transport by GLUT1 [31]. Failure in glucose transport and subsequent metabolic derangement can affect various functions of CPE cells, including CSF production, transport across BCSFB, and the secretion of growth factors into CSF [27]. However, the values of CSF glucose in patients with AD (*n* = 9) were not found to significantly differ from those in cognitively unimpaired individuals (*n* = 122) [32]. NMR metabolomics, such as glucose, of CSF distinguishes between patients with PD (*n* = 10) and controls (*n* = 10) [33].

#### 2.1.2. Fructose

Serum fructose concentrations in 23 healthy individuals, 26 individuals with diabetes, and 23 non-diabetic individuals were reported to be approximately 8.1 ± 1.0, 12.0 ± 3.8, and 7.7 ± 1.6 μM, respectively [34]. Serum fructose levels in healthy humans consuming high-fructose or high-sucrose diets can reach 200–500 μM [35]. However, fructose concentration in CSF is in the order of 100 μM, exceeding the plasma fructose level [36,37]. The physiological function of fructose in the brain is not fully understood. It is considered that fructose may act on a nutrient sensor in *Drosophila* [38]; however, it remains to be clarified whether fructose plays a similar role in nutrient sensing in mammalian brains.

A representative transporter for fructose, GLUT5, is expressed on the apical side of CPE and ependymal cells (Figure 2i) [39]. Another transporter for fructose, GLUT8, is also expressed in CPE cells [40]. Interestingly, fructose concentrations in CSF are higher than in serum. Adult rat and human CP transcriptomic analyses revealed low expressions of *Khk* and *Aldoc* [41], which are genes involved in fructose catabolism via the fructose-1-phosphate pathway [42]. These findings suggest that CPE cells may use fructose for energy production. Assuming that fructose is transported from CSF to CPE cells, a decrease in GLUT5 expression in CPE cells could cause an increase in CSF fructose concentration and result in the impairment of periventricular structures, including the hippocampus. Accordingly, changes in GLUT5 expression during aging and in neurodegenerative diseases may affect periventricular structures.

Regarding the association between CSF fructose levels and the pathogenesis of neurodegenerative diseases, some findings have been reported. A non-targeted and mass spectrometry-driven approach showed that CSF levels of fructose, as well as mannose and threonic acid, were significantly higher in 34 patients with early-stage Parkinson’s disease (PD) than in 35 healthy controls [43]. In addition, gas chromatographic/mass spectrometric and enzymatic methods applied to CSF samples from 85 patients with multiple sclerosis (MS) showed that concentrations of fructose, lactate, and sorbitol were significantly increased in the CSF of 54 patients with secondary progressive MS and, to a lesser degree, 31 patients with relapsing-remitting MS compared with 18 controls [44]. In addition, CSF concentrations of sorbitol and fructose correlated positively with the neurological disability score in patients with MS. Accordingly, fructose levels in CSF could be a useful marker for understanding medical conditions, such as exacerbations or remissions, in patients with MS.

#### 2.1.3. Urate

The normal serum reference levels of uric acid are 2.5–7.0 mg/dL (149–417 μM) in men and 1.5–6.0 mg/dL (89–357 μM) in women [45], whereas CSF urate concentration is 10- to 20-fold lower than in plasma [46,47]. The urate concentration in rat CSF was found to be higher than in rat brain ISF [48]. Moreover, the urate level was increased in the hippocampus in rats fed a high uric acid diet [49], suggesting the presence of a transporting system of dietary uric acid into the brain. Urate transporters in CPE cells are shown in Figure 2j–l [5,50]. Breast cancer resistance protein (BCRP)/ATP-binding cassette transporter G2 (ABCG2) is the main urate transporter at the blood–brain barrier (BBB), and it is expressed on the luminal membrane of capillary endothelial cells and considered to excrete brain urate into the blood [51,52]. Some studies revealed that URAT1, GLUT9, and BCRP are all expressed in the CPE and/or ependymal cells of human and mouse brains [50,51,53]. These results suggest that urate is transported from the blood to CSF via CPE cells and then from CSF to the brain parenchyma via ependymal cells.

Interestingly, an inverse association between serum urate levels and the risk and progression of neurodegenerative diseases, including AD, vascular dementia, and PD, has been reported [54,55], suggesting a causal neuroprotective effect of high urate levels. Bowman et al. [46] reported that CSF and plasma uric acid concentrations were positively correlated in thirty-two patients with mild-to-moderate AD. In addition, BBB impairment was associated with higher CSF levels of uric acid. CSF uric acid concentrations were independent of age, sex, and AD severity [46]. However, Tohgi et al. [56] reported that CSF urate concentration was significantly increased in patients with vascular dementia of the Binswanger type (VDBT) but significantly decreased in patients with AD compared with controls. They concluded that the significant increase in the CSF concentration of urate in VDBT is mainly due to BBB impairment and that the significant reduction in AD may reflect impaired brain metabolism [56]. These findings suggest that higher CSF levels of uric acid are associated with vascular disorders with BBB impairment.

#### 2.1.4. Lactate

The CSF lactate level has been reported to be 1.3–2.4 mM [57]. The lactate transporters in CPE cells are shown in Figure 2m,n [58]. In 7614 individuals, CSF lactate values were reported to be age dependent [29]. CSF metabolomics using proton nuclear magnetic resonance (NMR) spectroscopy of samples from 81 participants showed that the levels of lactate, as well as the levels of alanine, citrate, creatinine, leucine, tyrosine, and valine, significantly increased in older participants compared with those in younger ones [59].

A CSF-based study showed that lactate levels in patients with PD (*n* = 101) increased compared with controls (*n* = 60) and were correlated with both clinical disease progression and neurodegeneration biomarkers, such as tau proteins and dopamine [60]. Accordingly, lactate levels in CSF may be useful to help understand the degree of aging and disease progression of PD. In addition, the CSF samples of patients with MS showed that the concentrations of lactate, as well as fructose, were significantly increased in the CSF of 54 patients with secondary progressive MS and, to a lesser degree, 31 patients with relapsing-remitting MS compared with 18 controls [44]. However, there were lower levels of CSF lactates in patients with AD (*n* = 92) and frontotemporal dementia (FTD) (*n* = 27) than in individuals without dementia (*n* = 28) [61].

### 2.2. Dopaminergic and Serotonergic Neurotransmitter-Related CSF Metabolites

It is known that the neurotransmitter metabolites of dopaminergic neurons in the CSF of PD are diagnostically useful [62]. CSF 5-hydroxyindoleacetic acid (5-HIAA), the main serotonin metabolite, was found to be decreased in patients with PD and MSA, whereas CSF 5-HIAA levels in patients with PSP and corticobasal syndrome (CBS) did not differ from those of the control group [63].

Changes in glucose- and neurotransmitter-related CSF compounds in some disorders are summarized in Table 3.

## 3. Changes in CSF Compounds Caused by Neurodegeneration

### 3.1. Alzheimer’s Disease-Related Substances

Several kinds of age-associated CP alterations are prominent in AD, and they are considered to be linked to an increasing Aβ burden in CP [28,64,65]. The findings suggest that CP alterations in AD affect CSF components. A systematic review and meta-analysis of 231 articles comprising 15699 patients with AD and 13018 controls showed that 42 amino-acid-long amyloid-β peptide (Aβ1-42), total tau (T-tau), and phosphorylated tau (P-tau) are surrogate biomarkers of AD [66]. In addition, a study on CSF biomarkers involving 114 patients with AD also showed that Aβ1-42, T-tau, and P-tau are appropriate surrogate biomarkers of AD pathology [67]. As synaptic dysfunction is linked to AD, synapse protein concentrations in CSF may be useful biomarkers to monitor synaptic dysfunction and degeneration in AD. CSF neurogranin, a post-synaptic protein, has emerged as a promising tool to measure synaptic dysfunction and loss in AD [68,69]. A retrospective study showed that increased levels of neurogranin in patients with AD (*n* = 33) were significantly correlated with T-tau, P-tau, and the mini-mental state examination score [69]. A systematic review and meta-analysis with a narrative synthesis study showed that CSF neurogranin predicts mini-mental state examination decline in patients with amyloid-β mild cognitive impairment [70]. In addition, CSF neurogranin could be used to predict declines in memory and executive functions in the presence of mild cognitive decline. CSF neurogranin/Aβ1-42 ratios are also likely to help predict cognitive decline [70]. It was recently reported that the level of neuroligin-1 (Nlgn1), a post-synaptic cell adhesion protein, was reduced in the CSF of patients with AD (*n* = 43) compared with controls (*n* = 42) [71], indicating that Nlgn1 is an interesting synaptic biomarker candidate for neurodegenerative diseases. Amyloid-β oligomers, consisting of 10–20 monomers (AβO10-20), accumulate in the CSF of patients with idiopathic normal pressure hydrocephalus (iNPH) and are eliminated by CSF shunting, indicating that AβO10-20 may be an applicable diagnostic and prognostic biomarker of iNPH [72].

Recently, many data on changes in the expression of microRNAs (miRNAs), which are small and non-coding RNAs enriched in exosomes, in the CSF of patients with neurodegenerative diseases were summarized in a review paper [73]. In the review paper, miR-16, miR-17, miR-20a, miR-101, miR-106a, miR-106b, miR-147, miR-153, and miR-520c are introduced as miRNAs targeting the mRNA of amyloid precursor protein (APP). In addition, many miRNAs related to tau protein, neuroinflammation, and synaptic function have been reported [73]. Accordingly, it is considered that the expression levels of some miRNAs are closely associated with the pathophysiology of various neurodegenerative diseases, including AD. Variations in their levels could be diagnostic biomarkers for neurodegenerative diseases. Altered expressions of miR-320a, miR-328-3p, and miR-204-5p have been reported in AD (*n* = 28) and FTD (*n* = 12) [74]. Expressions of these three markers were found to be significantly lower in patients with AD than in controls (*n* = 8). Lower miR-328-3p levels could differentiate patients with AD from patients with FTD and controls and showed a significant correlation with lower Aβ1-42 levels. These findings suggest that miR-328-3p is involved in the AMPK signaling pathway, which is linked to amyloid-β and tau metabolism in patients with AD. However, it remains to be clarified which gene is regulated by the miRNAs. Various types of information on miRNAs may allow for the effective treatment of multifactorial diseases. Accordingly, the precise mechanism of miRNA–target interactions and the regulation of the targets by miRNAs should be clarified to develop successful miRNA-based drugs for the treatment of AD. In addition, recently, Hoshi et al. [75] reported that the mannosylated glycan structures of transferrin could be a new biomarker for AD. Table 4a presents the CSF compounds showing increased or decreased expression in the CSF of patients with AD.

### 3.2. Parkinson’s Disease and Other Neurodegenerative-Disease-Related Substances

CSF total α-synuclein has been studied as a surrogate biomarker of synucleinopathies [77,78]. Most studies agree that CSF total α-synuclein is decreased by 10–20% in patients with PD compared with healthy subjects and those with other neurodegenerative diseases. Phosphorylated Ser129 (pS129-) and oligomeric α-synuclein species are known to underlie and drive the neurodegenerative process in synucleinopathies [81,82]. A study on CSF α-synuclein species in 135 patients with neurodegenerative diseases [79] showed that patients with PD (*n* = 13) exhibited higher pS129-α-synuclein/α-synuclein ratios than patients with FTD (*n* = 26). In addition, a systematic review of CSF biomarkers for PD involving at least 20 patients with PD showed that oligomeric α-synuclein might be helpful in the separation of patients with PD from controls [62]. Patients with multiple system atrophy (MSA) (*n* = 9) were found to have lower α-synuclein levels than patients with corticobasal degeneration (CBD) (*n* = 9). Patients with a synucleinopathy (PD and MSA) exhibited lower total α-synuclein levels and higher pS129-α-synuclein/total α-synuclein ratios than patients with tauopathies (progressive supranuclear palsy (PSP) and CBD).

Delaby et al. [86] evaluated the differences in CSF neurofilament light chain (NfL) protein in the presence of various neurodegenerative diseases. Patients with neurodegenerative diseases, such as FTD, CBS, and PSP, showed increased levels of CSF NfL protein, suggesting increased neuroaxonal degeneration. It is reasonable to consider that CSF levels of NfL are useful to distinguish PD from other neurodegenerative diseases [62]. In addition, Schulz et al. [80] systematically assessed 10 biomarker candidates, such as total α-synuclein, NfL, phosphorylated neurofilament heavy chain, tau protein, ubiquitin C-terminal hydrolase L1, glial fibrillary acidic protein, calcium-binding protein B (S100B), soluble triggering receptor expressed on myeloid cells 2, and chitinase-3-like protein 1, in the CSF of patients with α-synuclein-related disorders. Among seven candidates showing a significant decrease or increase in expression, CSF NfL levels most effectively discriminated patients with PD (*n* = 151) and patients with MSA (*n* = 17) from controls (*n* = 20). CSF S100B most favorably discriminated patients with PD and dementia with Lewy bodies (DLB) (*n* = 45) from controls (*n* = 20). Rojas et al. [88] reported higher CSF NfL and lower phosphorylated tau concentrations with greater disease severity in patients with PSP (*n* = 50), indicating the usefulness of the CSF NfL/phosphorylated tau ratio for the diagnosis of PSP. In addition, the CSF levels of chromogranin A in patients in PD (*n* = 119) and MSA (*n* = 18) groups tended to be lower than those in a control group (*n* = 31), but there was only a significant difference between MSA and control groups [87]. CSF neurogranin is also decreased in patients with PD (*n* = 157), PD with dementia (*n* = 29), DLB (*n* = 11), MSA (*n* = 26), and PSP (*n* = 21) compared with controls (*n* = 47) and patients with AD (*n* = 124), emphasizing the importance of synaptic dysfunction in these parkinsonian disorders [83]. The expression of 17 miRNAs was reported to be upregulated in the CSF of patients with PD (*n* = 57) compared with controls (*n* = 65) [84]. Recently, miRNAs in the CSF of 11 patients with PSP were examined compared with controls (*n* = 8). miR-204-3p, miR-873-3p, and miR-6840-5p were most significantly up- or down-regulated in a PSP early-onset group [89]. The results suggest that miRNAs may be leading candidates as biomarkers of PSP. The target genes of these miRNAs were associated with molecules related to the ubiquitin–proteasome system and autophagy pathway. Table 4b,c present the CSF compounds showing increased or decreased expression in the CSF of patients with PD and Parkinsonism.

Recently, there has been increasing evidence showing that viable mitochondria in extracellular fluids act as an inflammatory signal [90]. In addition, circulating cell-free mitochondrial DNA (ccf-mtDNA) has been detected in the CSF of healthy individuals and patients with neurodegenerative diseases [91]. Podlesniy et al. [76] reported that a low content of ccf-mtDNA in CSF may be a novel biomarker for the early detection of AD, whereas Lowes et al. [85] reported that ccf-mtDNA levels were significantly reduced in patients with PD. Data on ccf-mtDNA will be useful for elucidating the mechanism of the pathogenesis in several kinds of neurodegenerative diseases.

## 4. Conclusions

In this paper, we first showed the significance of CP as the production site of CSF and the transport of intravascular nutrients and metabolized substances into CSF mainly based on papers recently published. As CSF is mixed with ISF, including substances physiologically and abnormally metabolized in neurons and glia through two major drainage pathways (the IPAD pathway and the glymphatic system) and via the ependymal cell layer, the analyses of CSF may be useful to explore biomarkers in order to diagnose neurological diseases. As summarized in Table 3, concentrations of glucose, fructose, and lactate in CSF can be useful to detect the pathological conditions of PD and/or MS, whereas urate concentration may be associated with vascular disorders, including VDBT. Neurogranin and neuroligin-1, post-synaptic proteins; the well-known surrogate biomarkers of AD, such as Aβ1-42 and T-tau; and P-tau are also useful for the diagnosis of AD. NfL protein, a cytoskeletal protein; total, oligomeric, and phosphorylated α-synuclein; and dopaminergic and serotonergic metabolites may also be useful for the diagnosis of PD. In addition, there is increasing evidence suggesting that the expressions of miRNAs and mitochondrial DNA in CSF increase or decrease in patients with AD, PD, and other neurodegenerative diseases. The accumulation of additional data on miRNAs and mitochondrial DNA in CSF is awaited to clarify the pathogenesis of various neurodegenerative diseases. As non-disease-specific substances due to neuronal death and synaptic dysfunction also affect components of CSF, an evaluation of multiple biomarkers in CSF may be useful to help understand the pathogenesis and progression of neurodegenerative diseases.

## Figures and Tables

**Table 1:** Transporters supposed to be involved in CSF secretion in CPE cells.

Molecules	Apical Side	Basal Side
H_2_O	AQP1	AQP1
Na^+^, K+	Na^+^−K^+^−ATPase	
Na^+^, K^+^, 2Cl^−^	NKCC1	
Na^+^, H^+^	NHE1	
Na^+^, HCO3^−^	NBCe2	NBCn1
Na^+^, Cl^−^, HCO3^−^		Ncbe
Cl^−^, HCO3^−^		AE2
Cl^−^	Clir, VRAC	
K^+^	Kir7.1, Kv	

Localization of transporters suggested to be involved in CSF secretion in CPE cells is shown. The water channel AQP1, Na^+^-K^+^-ATPase, Na^+^, K^+^, 2Cl^−^ cotransporter NKCCl, acid/base transporters NHE1 and NBCe2, Cl^−^ channels Clir and VRAC, and K^+^ channel Kir7.1 and Kv are expressed in the luminal membrane. Some acid/base transporters are expressed in the basolateral membrane: the Na^+^-dependent HCO_3_^−^ transporter NBCn1, Na^+^-dependent Cl^−^/HCO_3_^−^ exchanger Ncbe, and the anion exchange protein AE2. AQP1 is expressed in large quantities in the luminal membrane, whereas it is also in the basolateral membrane with a lower abundance.

**Table 2:** The concentration of ions and osmolality in CSF from animals.

Ions and Osm.	Rabbit ^4,7^	Dog ^7^	Human ^7^	Human ^8^
Na^+^, mEq/L	149	151	147	137 ± 1.8
K^+^, mEq/L	2.9	2.98	2.9	2.8 ± 0.1
Cl^−^, mEq/L	130	132.5	113	122 ± 1.9
pH	7.27	7.42	7.31	7.43 ± 0.02
Osm. (mOsm/L)	305.2	305.2	289	n/a

^4^: data reported in the review by Damkier and Praetorius [4]. ^7^: data reported in the review by Davson et al. [7]. ^8^: data expressed as mean ± standard deviation of non-diabetic control subjects (Age: 44.7 ± 14.0 year) reported in the original paper by Liao et al. 2021 [8]. n/a: not available, Osm.: Osmolality.

**Table 3:** Glucose- and neurotransmitter-related CSF metabolites of disorders.

	CSF Compounds	Related Disorders	Expression	Cited Papers
(a-1)	glucose	aging	inc	[29]
		diabetes mellitus	inc	[30]
		bacterial meningitis	dec	[31]
		Parkinson’s disease	dec	[33]
(a-2)	fructose	Parkinson’s disease	inc	[43]
		multiple sclerosis	inc	[44]
(a-3)	urate	BBB impairment	inc	[46]
		VDBT	inc	[56]
(a-4)	lactate	aging	inc	[29,59]
		Parkinson’s disease	inc	[60]
		multiple sclerosis	inc	[44]
(b-1)	dopaminergic	Parkinson’s disease	dec	[62]
	metabolites			
(b-2)	5-HIAA	Parkinson’s disease	dec	[63]
		MSA	dec	[63]

inc: increased expression, dec: decreased expression. BBB: blood-brain barrier, 5-HIAA: 5-hydroxyindoleacetic acid. MSA: multiple system atrophy, VDBT: vascular dementia of the Binswanger type.

**Table 4:** CSF compounds of neurodegenerative diseases.

Diseases		Related CSF Compounds	inc/dec	Cited Papers
(a)	Alzheimer’s	amyloid-b (1-42)	dec	[66,67]
	disease	total tau	inc	[66,67]
		phosphorylated tau	inc	[66,67]
		neurogranin	inc	[68,69]
		neuroligin-1	dec	[71]
		microRNAs	inc/dec	[73,74]
		Man-transferrin	inc	[75]
		ccf-mtDNA	dec	[76]
(b)	Parkinson’s	total α-synuclein	dec	[77,78,79,80]
	disease	p-α-synuclein	inc	[79,81]
		oligomeric a-synuclein	inc	[62,82]
		NfL	n.s./inc	[62,80]
		S100B	dec	[80]
		neurogranin	dec	[83]
		microRNAs	inc/dec	[84]
		ccf-mtDNA	dec	[85]
(c)	Parkinsonism	α-synuclein	dec	[77,78,79,80]
		NfL	inc	[80,86]
		neurogranin	dec	[83]
	(DLB)	S100B	inc	[80]
	(MSA)	chromogranin A	dec	[87]
	(PSP)	NfL/p-tau ratio	inc	[88]
		microRNAs	inc/dec	[89]
	(iNPH)	Ab oligomers (10-20)	inc	[72]

inc: increased expression, dec: decreased expression, inc/dec: increased or decreased expression, n.s./dec: not significant or decreased, ccf-mtDNA: circulating cell-free mitochondrial DNA, DLB: dementia with Lewy bodies, MSA: multiple system atrophy, Man-transferrin: mannosylated-glycan transferrin, iNPH: idiopathic normal pressure hydrocephalus, NfL: neurofilament light chain, p-α-synuclein: phosphorylated α-synuclein, PSP: progressive supranuclear palsy, S100B: calcium-binding protein B.

## Abbreviations

Aβ1-42	amyloid-β(1-42)
ABCG2	ATP-binding cassette transporter G2
AD	Alzheimer’s disease
AE2	anion exchange protein 2
APP	amyloid precursor protein
AQP1	aquaporin 1
BBB	blood–brain barrier
BCRP	breast cancer resistance protein
BCSFB	blood–cerebrospinal fluid barrier
CBD	corticobasal degeneration
CBS	corticobasal syndrome
Ccf-mtDNA	circulating cell-free mitochondrial DNA
CP	choroid plexus
CPE	choroid plexus epithelial
CSF	cerebrospinal fluid
DLB	dementia with Lewy bodies
FDG	fluorodeoxyglucose
FTD	frontotemporal dementia
GLUT	glucose transporter
HIAA	hydroxyindoleacetic acid
iNPH	idiopathic normal pressure hydrocephalus
IPAD	intravascular periarterial drainage
ISF	interstitial fluid
miRNA	microRNA
MS	multiple sclerosis
MSA	multiple system atrophy
NfL	neurofilament light chain
Nlgn1	neuroligin-1
NMR	nuclear magnetic resonance
PD	Parkinson’s disease
PSP	progressive supranuclear palsy
P-tau	Phosphorylated tau
SGLT2	sodium/glucose cotransporter 2
S100B	calcium-binding protein B
T-tau	total tau
VDBT	vascular dementia of the Binswanger’s type
